# Analysing environmental opinion using highly customisable visualisation tools to understand citizens’ attitudes and barriers

**DOI:** 10.1038/s41598-024-54457-3

**Published:** 2024-02-16

**Authors:** Luz Calvo, Marta Terrado, Mario Pérez-Montoro, Diana F. Vélez, Fernando Cucchietti

**Affiliations:** 1 Barcelona Supercomputing Center, CASE, 08034 Barcelona, Spain; 2 Barcelona Supercomputing Center, Earth Sciences, 08034 Barcelona, Spain; 3 Faculty of Information and Audiovisual Media at the University of Barcelona, Melcior de Palau, 140, 08014 Barcelona, Spain

**Keywords:** Data visualisation, Decision support system, Cognitive load, Usability, Environmental opinion, Service design, Interaction, Climate-change policy, Energy and society, Environmental impact, Psychology and behaviour, Socioeconomic scenarios

## Abstract

Knowledge of public opinion is key to understanding citizens' attitudes towards environmental policies. However, large polls and surveys generate complex datasets from which it is not always easy to draw conclusions. In addition, tailor-made solutions for analysing public opinion face the challenge of handling too many layers of information, which can easily lead to an overwhelming user experience and impair decision-making. Service design methodologies can support the design of ad hoc visualisation tools focused on user needs. We present *Op-e-nion*, a case study of a visualisation tool for the analysis of public opinion regarding environmental issues, aimed at administrations and public institutions. The involvement of experts from different fields allowed for the identification of the main metrics necessary to target the least engaged socio-demographic groups as well as the barriers that limited their environmental actions. Experts also highlighted useful aspects of the design process and the final prototype to help them define more effective campaigns and policies to address social challenges and promote citizen action. An innovative step was introduced in the methodology by involving non-state actors in the evaluation of the tool, ensuring problem detection and enhancing the sustainability of the final product. Important aspects for the visualisation of multi-categorical data included simplifying the interaction with the tool while prioritising relevant information, and using highly customizable visualisations to answer specific user requirements and changing needs (i.e. analytical vs. managerial tasks). Improved visualisations of public opinion data will, in turn, better support the development of policies shaped by citizens’ concerns.

## Introduction

Public participation plays an important role in efforts designed to understand social issues. However, to achieve the expected goals, this participation must be defined in a meaningful way for both citizens and administrative institutions^1^.

Knowledge of public opinion allows policymakers to understand social trends and attitudes provided that widespread participation is achieved^2^. It also helps them predict the actions and responses of the population to certain social issues, as well as to understand the behaviour or criteria of some groups or collectives^3,4^. This knowledge offers environmental organisations and public administrations information on the opinions, degree of satisfaction, and concerns of citizens^5^.

Recent studies show how social attitude analysis and social networks can be used to influence or even alter citizens' responses for the benefit of private interests^6^. However, these social media advertisements and campaigns can also be used to educate segments of the population in the face of global challenges and to favour progress and the common good, in relation to aspects such as sustainability, socially inclusive policies, equality, and resource optimisation^7,8^.

The analysis of open and multi-categorical data allows for the extraction of highly valuable information and for the identification of patterns in the behaviour, beliefs, concerns, and attitudes of citizens. However, interpreting complex datasets poses new challenges in the field of data visualisation^9^. Indeed, the geographical representation of social multi-categorical data has some limitations due to the inherent nature of maps, which involve a spatial overlap of information and a need for interaction^10^.

The comprehension of social patterns for different levels of geographic granularity (continent, country, region, city, neighbourhood) is a global need for governments^11^. However, the tools available to understand geocodified information, based on geographic information systems (GIS) are often too complex for non-expert users^12,13^ (see also Table 1 of the Supplemental Material).

**Table 1:** Positive and negative comments gathered during the Bipolar Laddering test along with the number of times each aspect was mentioned (frequency) and the average value it received.

Description	Freq.	Avg.
**Positive aspects**		
Usefulness of visualisation customisation for the different views and depending on the task of interest	12	9.7
Presentation of inconsistencies usefulness: Ability to save time and help reaching conclusions	11	9.1
Comparison of socio-demographic groups usefulness and intuitiveness	8	9.4
Interactions/Exploration are intuitive and clear: the user knows how to interact at each moment	7	9.4
Information density and amount of insights gathered are presented in a clear and familiar way	6	7.3
Contrast with dark background is very clear	5	8.4
Summary view is very useful as a complement to the general overview	5	8.4
Simplicity of the representations	5	8.2
Utility of charts, step-by-step information and level of deep analysis	2	8
Totals by region/category usefulness for checking general insights of the area/region under analysis	1	7
Global view represented as concentric circles gives a lot of information and works well with problems of overlapping	1	8
*Total*	**63**	**8.4**
**Negative aspects**		
Complexity of the global visualisation using concentric circles summarising all opinions for all socio-demographic groups	7	8.2
Vague legend descriptions in the initial representations: Information about the diameter size of the concentric circles is needed	6	4.4
Inverted scale (darker colours for lower values) seems strange until you understand the user requirements	4	5.2
Showing all labels for all socio-demographic groups simultaneously	3	4.6
The sorting by negative aspects seems strange until you understand the importance of highlighting the less committed groups	2	4.5
The dots to indicate inconsistencies in one socio-demographic group are too visible	2	2.5
Some labels in the summary view must be relocated to a different place	1	4
Detailed glyphs are complicated at first sight	1	4
Comparison of the status of socio-demographic groups is not intuitive	1	6
Help or more detailed information is needed at some points/sections	1	3
Not enough contrast for some figures	1	4
*Total*	**29**	**4.6**

In addition, visualisations should go beyond simply showing data or offering mechanisms for interaction through which administrative bodies and institutions can find specific information. A truly useful tool must also guarantee the understanding of the information, highlight relevant aspects, favour conclusions and comparison, as well as support the user’s decision making mechanisms^14–17^. To achieve these goals, it is necessary to involve experts from diverse disciplines (e.g. natural and social sciences, visualisation, user experience) that can help define effective representations of complex high-density data^18^. On the other hand, to be able to accommodate differing user profiles, levels of expertise and specific and changing needs, the user should also be able to customise the way the information is displayed^19^. This additional flexibility allows users to better understand the data and explore it on their own terms^20^.

Focusing on the highly customizable visualisation analytical tool *Op-e-nion*, this paper includes the following contributions:

- Overview on the importance of the analysis of public opinion and attitudes to promote more effective social policies (Section “Previous work”).
- Identification of the most relevant information for administrative institutions using public opinion datasets. In this case, we use the environmental dataset gathered by the European Union^21^, in order to design specific and targeted campaigns to promote action from citizens. However, this step is extendable to all types of public opinion analysis with multi-categorical data at different geographical levels (country, community, region, and even urban).
- Delineation of primary analysis tasks relevant to administrative institutions when analysing public opinion at a socio-demographic level (Section “Public open data on environmental issues”);
- Description of the main features included in *Op-e-nion*, along with a discussion of the service design process (Section “Tool functionalities”);
- Importance of involving experts from different fields in the visualisation design process and the application of a two-step evaluation for improvement of the visualisation (Sections “Conceptualisation” and “The importance of evaluating the tool from different perspectives”);
- Importance of offering a high level of chart customisation and visualisation included within the tool to tackle changing and specific needs for different types of users to support decision making processes (Section “The importance of flexibility and customisation in decision making”);
- Adding Bipolar Laddering and NASA-TLX methodologies to reinforce user testing results. Both techniques are described in the Methodology section. The first helps to evaluate the usefulness of functionalities independently of the way they were implemented, and the second one helps to measure the cognitive load in users.
- As “information architecture (IA)” allows users to navigate or consume information step-by-step within a website, we have coined the term “visualisation architecture” or “chart architecture”, as the visualisation of the tool itself, together with the interaction design, acts as an element to favour deeper navigation of the visual information.
- Summary of the response of different types of users (i.e. end users of administrative institutions and non-state actors). We also discuss the importance of combining this response with feedback from different experts in the fields of service design, visualisation and graphic design for data visualisation, who can provide more detailed insight for the improvement of the tool (Section “The importance of evaluating the tool from different perspectives”).

## Previous work

### Visualising multi-categorical data of public opinion

When visualising survey and opinion results, the choice of the most suitable chart often depends on the question type (numeric, likert, multiple choice…)^22,23^ and the user expertise level^24^. Moreover, the charts used to represent the data gathered must be clear, precise, and familiar to the users^25^. Depending on the target audience, the use of familiar visualisations such as bar charts, line charts, donut charts or stacked bars is recommended^26^, but they are inefficient when comparing values of multiple charts^27^. The use of less conventional representations such as waffle charts, dot charts, radar charts or lollipop charts is no longer effective as more than two categories are represented simultaneously^23,26^. Regarding the use of colour, in the case of scales with more than two categories, it is advisable to use diverging colour scales (instead of the classic representation using qualitative colours), to indicate a higher or lower priority of the results presented^28,29^.

Effectively communicating multi-categorical data containing more than three dimensions is a challenge, not only from a visualisation point of view but also from a user experience perspective^30^. The most frequent existing alternatives combine different charts including information on the relationships between them and often involve multiple visual properties such as colour, size, position, shape, and the use of patterns and textures on multiple layers. However, when the number of categories is high, many of these representations lose their effectiveness and require evaluation from a perceptual point of view^31^. Moreover, the visualisation of multi-categorical data is closely linked to two key user tasks: classification and comparison. Therefore knowledge of user expectations and their search patterns is essential when proposing effective, significant and easy-to-understand visualisations^32,33^.

### The role of Visualisation in administrative tools for decision making

The influence of public opinion in the state and administrative policy design process is one of the most urgent goals in achieving effective policies^34,35^. The knowledge and analysis of public opinion data implies, in the first place, knowing the events that affect community sentiment and the citizens’ demand for public administration action. This knowledge allows them to learn of users’ interests, their level of trust in the administration and the mechanisms of education that contribute to direct and moderate the social response. Obtaining reliable data increases the effectiveness of the communicative dialogue between authorities and civil institutions^35^.

Despite the strong relationship between social research and public policy, the most prominent international surveys have little or no presence in public policies in some countries^34^. People's way of thinking, behavioural patterns and innovative management ideas, have a high potential in the application in the field of public administration. Access to this data is critical to many aspects of society. However, analysing this big data and defining how to use data visualisation to show and optimise social phenomena are the challenges that government administrations face^36,37^. The existing data visualisation tools often do not allow us to analyse, filter, compare or interact with data at a deeper level. In this sense, the analysis of specific cases can provide transparency about the use of data visualisation and technology, in order to promote more sustainable practices^38,39^.

**Figure 1:**
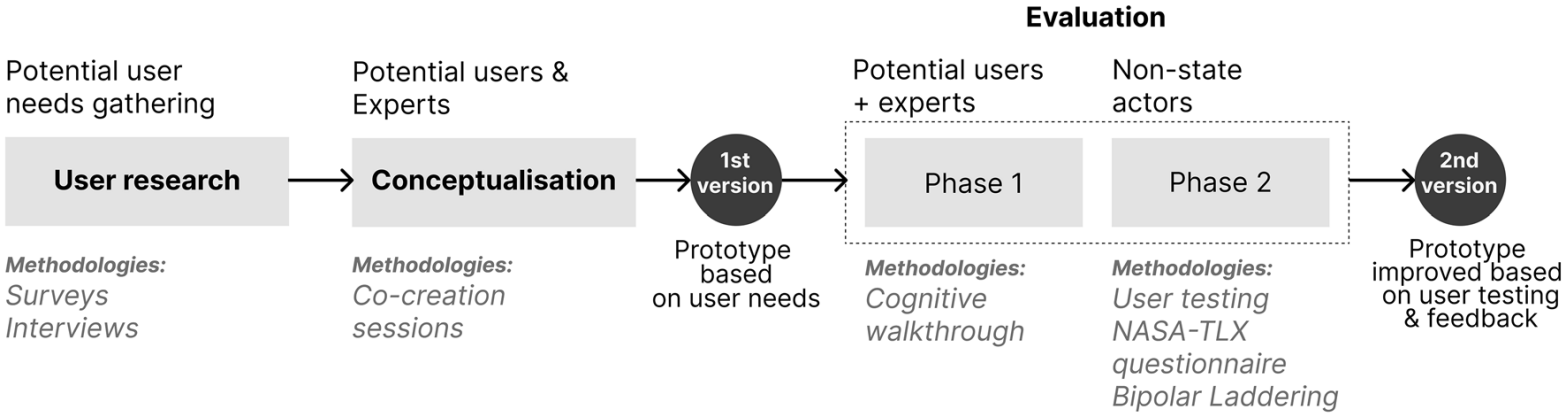
Service design approach applied to the design of a data visualisation tool, including user research, conceptualisation and evaluation. The evaluation stage was carried out in two phases, involving different users and methodologies and allowing the evolution from the first to the second version of the prototype.

The tools currently available to users of global public opinion datasets (e.g. Eurobarometer) as well as other tools focused on the analysis of public analysis^40–42^, only allow for the visualisation of answers by country, but not for the exploration of the answers at a socio-demographic level (see Fig. 1 of the Appendix).

This study applies a service design approach for the codevelopment of a tool tailored to the requirements of potential public administration staff, interested in designing effective policies and campaigns to promote citizen action. As a use case, we present the co-creation design process of the data exploration and visualisation tool *Op-e-nion,* focused on the analysis of citizen opinion in regards to the environment. During the process, we determine which kinds of variables and environmental aspects are more relevant for city councils and administrations, we design an initial prototype with the requested user functionality, and evaluate the prototype with potential users and experts as well as with non-state actors. We show how, through the use of interactive mechanisms and effective charts, together with highly customizable visualisations, we favour common analytical processes while identifying barriers behind opinion or attitudes towards public policy, not only on a global or country level, but also at a socio-demographic group level.

## Data source

For illustrative purposes, an open environmental dataset from the Eurobarometer database was selected as a use case and is described in “Visualising multi-categorical data of public opinion”. This type of data is often analysed in the public administration sector to learn about citizens’ behaviours and understand attitudes and actions towards the environment. The methodology applied for the design of the service, involving a user research, a conceptualisation and an evaluation stage, is then described in Sect. 3.2. This methodology approach enabled the development of a first version of the prototype based on user needs. This prototype was subsequently modified through user testing and feedback, resulting in a second, improved version.

### Public open data on environmental issues

Eurobarometer is a series of open datasets regarding public opinion on different topics of public interest and published by the European Commission and which are updated periodically (two or three years). Specifically, the selected dataset referred to the attitudes of ‘EU citizens towards the Environment’ collected in 2017^21^.

This dataset was selected because its structure matched the needs of the administrative users involved in the study, as it contained multi-categorical information on social opinion, extensive categorisation by socio-demographic groups (see Table A.3 and Appendix A of the Suppl. Material) and the same answer types (such as likert scales to open-ended questions or rankings of options) (see Tables A.1 and A.2 of Appendix A for further details).

The dataset includes information regarding the following aspects:

1. General attitudes towards environmental issues: first associations and main concerns.
2. Personal perspectives on the environment: attitudes and behaviour.
3. Opinions on environmental policy: acceptability of sustainable development approaches, preferred policies, support for a single European environmental policy and the role of the EU as its driver.
4. Information channels: sources of information, feeling of being informed and topics of interest for which a lack of information has been detected.
5. Answers broken down into different categories and socio-demographic groups (gender, age, income, civil status, level of education, etc.).

## Gathering and refining user requirements

A service design approach was adopted to guide the tool creation process. This approach allowed us to address the domain and visualisation challenges while favouring sustainable development^43,44^. The applied methodology included three main stages (see Fig. 1):

In the initial stage of research, requirements from potential users were collected and the relevant data from the Eurobarometer was identified. Once the research stage has been finalised, the conceptualisation stage was carried out, in which possible solutions were explored. At the same time, an assessment of currently available tools was carried out to explore alternative solutions.

Service design involves users and stakeholders at each stage of the process in a more meaningful way than other user-centred design methodologies, and addresses the design challenges from different angles and perspectives^46^. At the same time, this methodology helps to save efforts and resources (e.g. human, time, consumption), as it validates the tools, their viability and the value they bring to the end user, before the development process is carried out^44,47^. The following sections describe each stage of the process in more detail.

### User research

An initial survey was conducted to explore the general information needs of potential users. The objective of the survey was to identify the relevant information to be shown in the tool as well as the questions that potential users need to answer through the visualisation of the information.

They also identified the relevant socio-demographic groups to be included in the tool (see Table A.3 of the appendix). The final socio-demographic groups selected were based on their capability to help them model fictional characters to be presented in advertisements or educational campaigns and with which the audience could feel identified^48,49^.

In addition, individual interviews were run with the same individuals with the objective to delve into more specific government needs that affect the planning of social actions. Interviews also helped identify target groups, which in this case corresponded to those collectives that were less committed with environmental issues, with the final aim to motivate their participation, and/or find new ways for them to engage in environmentally-friendly actions.

### Conceptualisation

After the gathering of user needs, potential users and experts from various disciplines were invited to participate in two online co-creation sessions. With the help of the collaborative design tool MURAL^50^, several possible solutions were identified and discussed. These types of sessions are useful since they provide room for assessing various functionalities and representations. Potential users can also discuss the pros and cons of each alternative and possible new solutions and features that may arise^51,52^. In this particular case, the co-creation sessions contributed to a more detailed analysis of the problem and the common daily tasks described by potential users, the usefulness of the selected data, as well as the questions that the visualisation should inform about. These sessions also allowed us to design an initial prototype, which was later improved during the evaluation stage. At the same time, an exploratory data analysis (EDA) was carried out to verify the effectiveness of the suggested glyphs with real data. This exploratory analysis was performed in the R programming language^53^. EDA facilitates a deeper understanding of data structure, enabling informed decisions in designing visualisations that accurately represent the underlying information while uncovering actionable insights^54^. No data preprocessing was needed for the design of the tool.

## Tool functionalities

**Figure 2:**
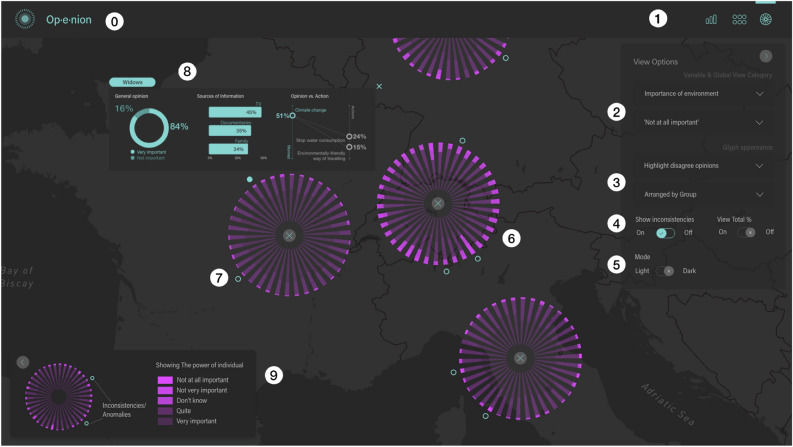
Op-e-nion tool dashboard in dark mode, with a detailed view of the socio-demographic groups by country. The main navigation (top bar) shows the three main options from left to right: Summary, Grid and Explore options views (1). On the right, a filter menu to select the variable and the type of answer to be represented (2) as well as the information to be highlighted (3). The option to show inconsistencies can be activated (4) and the user can select between the light and dark modes (5). Clicking on one of the inconsistency dots (7) opens a detailed view panel (8). Specific legends are shown for each of the different views (9).

The user research stage helped in the selection of the variables and demographics, as well as the functionalities and representations to be included in the first prototype of the Op-e-nion tool (presented in Fig. 2). Some of the main variables selected were related to the citizen empowerment, impact, and responsibility, as well as the EU policy obligations (see Table A.1 of Appendix A). The demographics selected from the original Eurobarometer dataset were organised in different categories (gender, age, socio-professional level, marital status, etc.) to represent the most significant groups and motivational situations for policy advertisement design (see Table A.3 of Appendix A).

The research and conceptualisation stages allowed us to define the main user requirements and questions these users need to answer:

1. Identify the less committed countries within the European Union and also the less committed socio-demographic groups in one or more countries. This was related to different indicators included in the dataset such as empowerment (individual and group), impact on health and environment, responsibility and EU policy obligations (see Appendix A).
2. Compare the level of commitment of a certain socio-demographic group (for example ‘Students’ or ‘Upper class’) among several countries.
3. Contrast the answers on the interests and concerns expressed by each group versus the environmental actions they actually carried out (we term this last requirement as ‘inconsistencies analysis’). This was a very challenging process due to the distribution of the information over several file tabs, and one of the most time-consuming tasks to be solved from the users’ point of view.
4. List the information channels (print media, digital media, television and radio, word of mouth, etc.) for those groups where inconsistencies were detected in order to address them through the channels which the users frequent the most.
5. Facilitate access to summary views aimed at managers in order to condense the relevant information.

Using the main navigation elements (see Fig. 2.1), users could choose how to access information, either in an exploratory manner (accessing data step-by-step and selectively) or by comparing different groups or areas. These options are typically associated with analytical user profiles. On the other hand, there is also the option to access summary views, from which the users can draw direct conclusions and export the visualisations to be used in reports and presentations (see Fig. 2b,c). Often, this option is more convenient for management profiles. Filtering options available in the tool (Fig. 2.2–5) allows users to select the information to be shown and how to represent it (i.e. the variables to be analysed, the answers users want to highlight and how they want to sort the results).

**Figure 3:**
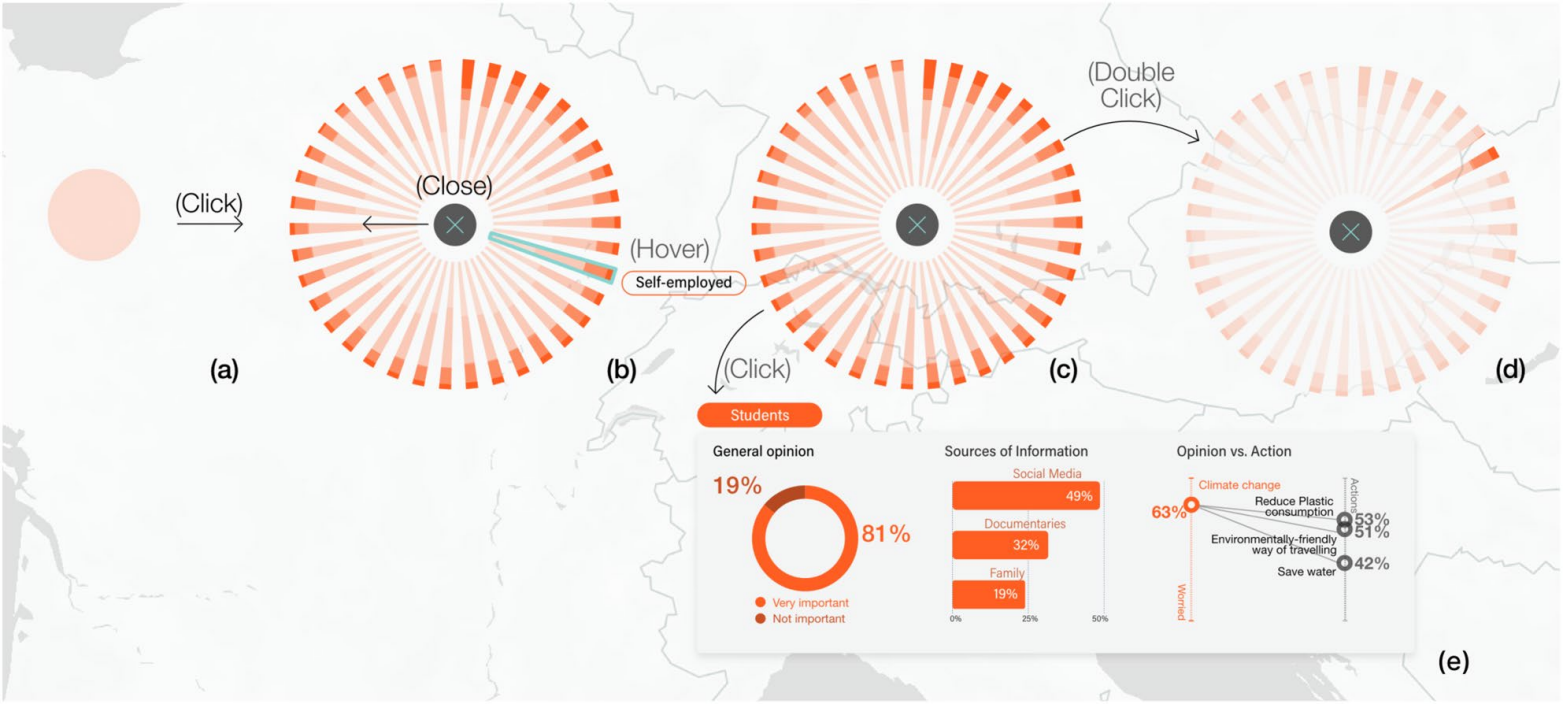
Accessing different levels of detail through interaction: By clicking on the general view (**a**), the user accesses the socio-demographic details. By hovering each category we can view the name of each group (**b**), and we access detailed information about rates by clicking on the element, together with sources of information. This is what we call the ’inconsistencies view’ (**e**). By double clicking on one of the groups, we enter the ‘Group comparison view’ (**d**).

**Figure 4:**
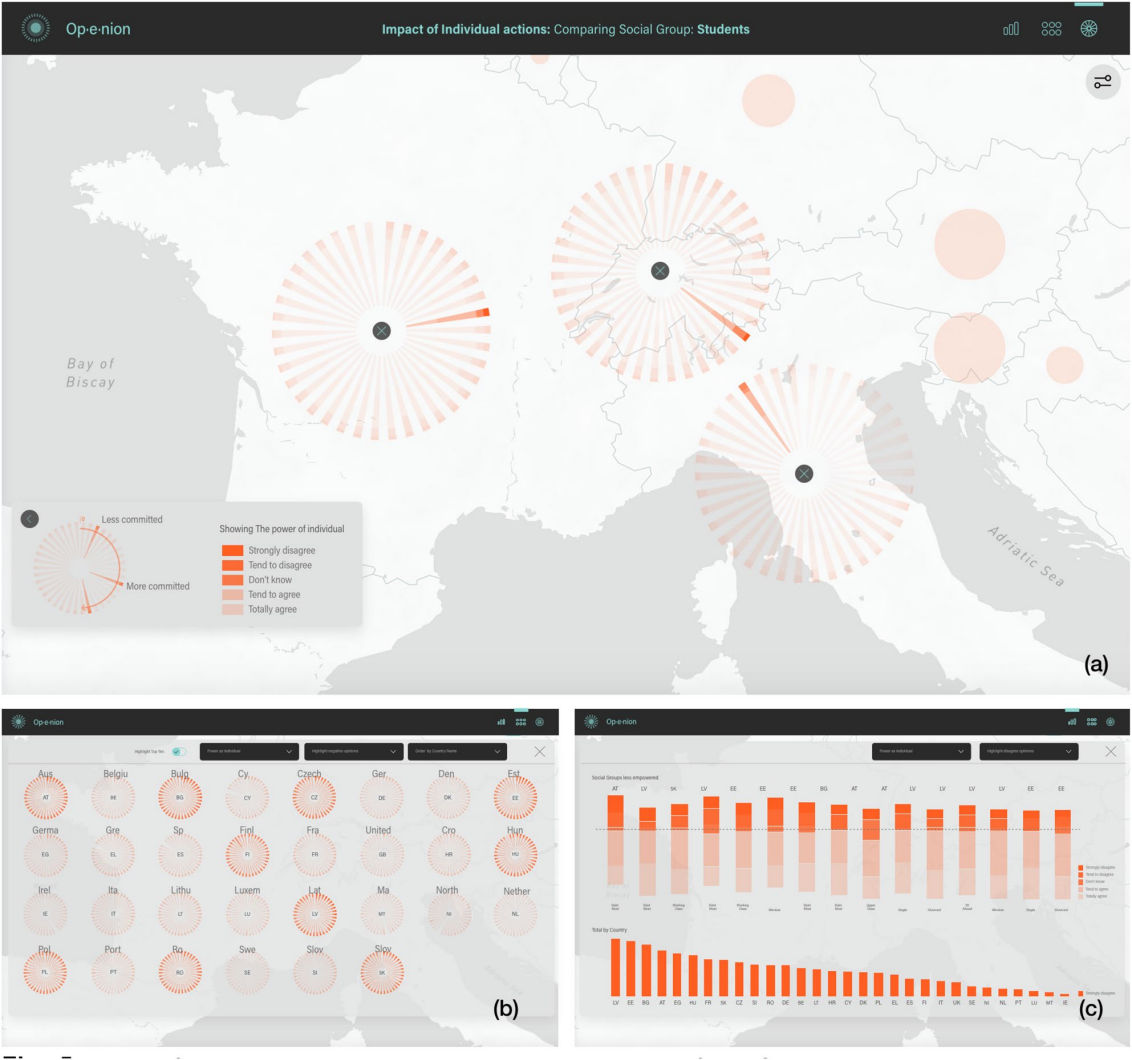
Detail of the explore options, grid and summary views of the Op-e-nion tool. Explore option view comparing the level of commitment between countries for a certain socio-demographic group, in this case ‘Students’ (**a**). Commitment levels are reflected using a ‘clock face’ interpretation, with higher commitment levels represented as later times. Grid view highlighting the top ten countries with higher values for the category ‘Totally disagree’ (**b**). Summary view with a ranking of the least committed groups with the environment in the European Union (**c**, top) and the sorting by negative opinions of the 30 least committed countries (**c**, bottom).

By interpreting the information step-by-step, different levels of detail are presented for each region. Thus, the general view presents glyphs with the total values for the different variables aggregated per country represented by simple bubble charts (See Fig. 3a). This view allows the user to explore geographic patterns such as the similarities and differences between neighbouring countries, or groups of countries sharing some characteristics (Eastern Europe, Central Europe, etc.). After identifying the most or the least committed countries or their attitude towards a specific environmental issue, users can access a more detailed view of the information according to the socio-demographic group (See Fig. 3b,c). A double click on a particular group, opens the comparative view where the group is highlighted for the different regions under consideration (See Figs. 4a, 3d).

**Figure 5:**
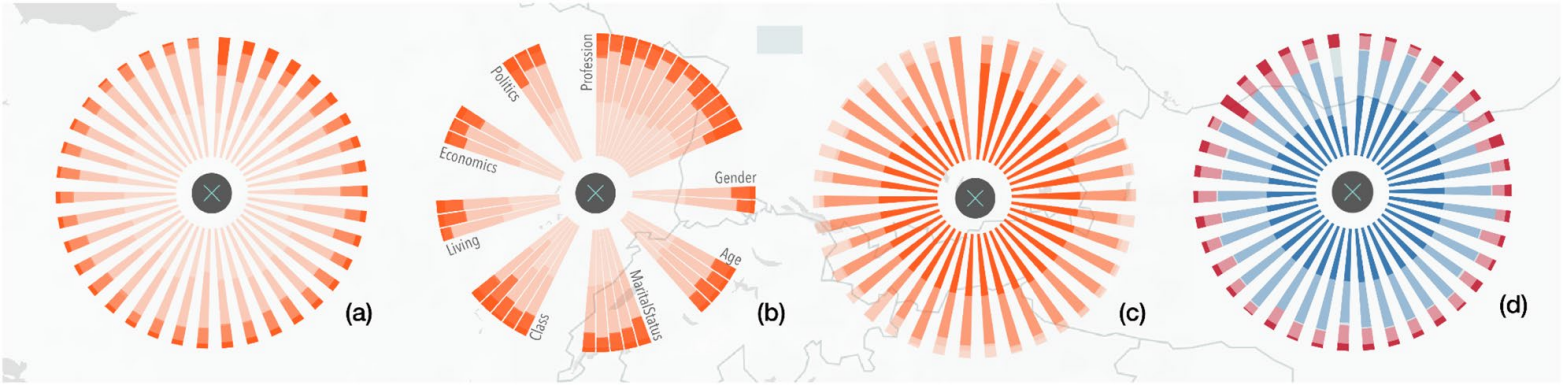
Four versions of the visualisation using radial stacked bars with detailed information on the responses of socio-demographic groups to the variable 'The role you can play as an individual'. Sorted by (and highlighting): the most negative answer 'Totally disagree' (**a**), group categories (gender, age, marital status, class, living area, etc.) (**b**), the most positive answer 'Strongly agree' (**c**), the most positive answer (**d**). Versions (**a**–**c**) use a sequential scale whereas version (**d**) uses a divergent scale.

The detailed glyphs for the different socio-demographic groups can be shown sorted in three different ways depending on the specific needs of the users (See Fig. 5): either highlighting the most positive or committed answers, highlighting the most negative or least committed answers or grouping them by categories (age, gender, income level, etc.). See Table A.3 of the Appendix for a complete list of the socio-demographic group categories. At the same time, we can use sequential scales (Fig. 5a–c) or divergent scales (see Fig. 5d). By clicking on one of the groups, we open a more detailed view (see Fig. 3e). By means of the detailed representation, users can also identify patterns between attitudes or concerns and action or involvement in social activities. By activating the inconsistencies view, the groups with conflicts between concerns and actions are highlighted (see inconsistency dots in Fig. 2.6,7). This reveals, for example, groups which claim to be concerned about environmental issues but which show a low level of commitment in carrying out actions to tackle them. Further details on the categories of information to define inconsistencies are available in Table A.2 of the Appendix. This view also enables the analysis for general answers’ ratios as well as the main communication channels used by these groups (see Fig. 2.8).

## Evaluation phases and methods

In the evaluation stage, the effectiveness of the tool was assessed through a two-step process. This included a first evaluation with potential and expert users and a second evaluation with non-state actors in order to guarantee the general usability of the tool^45^. The insights gathered during the qualitative and quantitative methods were analysed and considered in order to improve the final tool design (see Fig. 1 and section "Improvements applied based on evaluation results").

### Evaluation phase I: with potential users and experts

The first phase was carried out with potential users (five participants) who were also involved in the user research and conceptualisation stages. These two evaluation phases were especially important not only to check whether the proposed solution was adequate for the intended users. Participant profiles included city council staff, public entities, and stakeholders from public administration. Given that some of the participants were not used to working with tools and data at a national level, the evaluation of functionalities was performed by adapting the requirements to a potential application at the municipality, neighbourhood and district management levels.

The individual sessions were carried out using Cognitive Walkthrough (CW), a methodology used to test a product in a simulated usage situation^55,56^. Through CW, the participants could explore the tool freely, commenting aloud on the different issues they encountered, as well as suggesting improvements in terms of functionality.

*A* second round of individual sessions was also held with experts in the areas of data visualisation (three participants), user experience (two participants) and graphic design (one participant). These sessions generally focused on specific aspects of usability, graphic representation, data visualisation and their efficiency in communicating the particularities in the displayed data^57^*.*

### Evaluation (Phase II) with non-state actors

In this second phase of the evaluation, we sought to validate the ease of use of the tool with non-state actors (NGOs, firms, epistemic communities, etc.). These types of tests are carried out in order to detect problems related to the comprehension of the visualisations and their ability to aid comparison and decision-making^58–60^. Moreover, it was adequate for the intended users but also to validate that it made sense for a broader public.

The convenience sample consisted of twenty participants (eleven men and nine women of ages between 25 and 66) with low to medium knowledge of data visualisation^61^. Participants had not received any previous training and their fields of work encompassed administration, marketing and communication, environmental consultancy and independent activists. These users performed the sessions remotely due to COVID-19 restrictions.

We applied the user testing methodology^62^, starting with a brief explanation to provide participants with the necessary context, and then we asked them to solve five tasks presented in logical order of search and exploration. The tasks set out in the test aimed to reproduce the common daily tasks of potential users that had been identified during the user research stage (see Section "User research"). Each task was linked to a specific functionality and visualisation, indicated in parentheses at the end of the task description. We asked users to explore the prototype using the thinking-aloud technique^63,64^, while explaining to the moderator of the test exactly what they were thinking and what their main doubts were. In addition, we asked them questions to check their understanding of some specific aspects of the prototype.

**Task1:** Identify which countries have less interest in environmental issues (*General overview*).

**Task2:** Detect the least committed socio-demographic groups for one country (*Detailed view by country and socio-demographic group*) .

**Task3:** Compare the opinion (level of agreement, importance, etc.) on a certain question from one socio-demographic group for multiple countries (*Socio-demographic group comparison view*) .

**Task4:** Detect and analyse inconsistencies between concern and action (*Inconsistencies view*).

**Task5:** Analyse summary visualisation ranking charts for the least committed groups and countries (*Summary view*).

After the user testing, two additional activities were carried out in order to quantify the perceived cognitive load as well as the severity of the problems detected.

First, to assess the perceived difficulty and cognitive load, users completed a questionnaire for each task based on the NASA-TLX categories for different metrics: mental demand, temporal demand, frustration level and perceived effort^65^.

Second, for insight gathering we used the Bipolar Laddering methodology, in which users commented on the positive and negative aspects encountered while performing the tasks during the user testing. They rated these aspects from 1 to 10 to assess their usefulness or severity in a more precise fashion. The number of times that a specific aspect was mentioned by users gave an idea of its weight or relevance^66^ (See also Sect. C.3 in the Supplemental Material).

All methods and procedures outlined in this study were conducted in strict accordance with the relevant guidelines, protocols, and regulations pertaining to the field of UX Research and Service Design. The research was carried out with the utmost consideration for ethical standards and best practices.

The experimental protocols employed in this study were meticulously reviewed and approved by the Bioethics Committee of the Universitat de Barcelona (UB) prior to the commencement of any research activities. This approval underscores the commitment to adhering to rigorous scientific and ethical standards throughout the course of this investigation.

Informed consent, as per established ethical principles, was obtained from all participating subjects prior to their inclusion in this study. The consent process ensured that subjects were provided with comprehensive information about the study's objectives, procedures, potential risks, and benefits, and that they had the opportunity to ask questions and make an informed decision to participate. See more details of the user testing environment and participants in the Appendix B of the Supplemental Material.

## Evaluation results

### (Phase I) with potential users and experts

Results from the Cognitive Walkthrough (CW) show how potential users valued aspects such as the ability to compare all socio-demographic groups at the same time for different countries. Sections such as the summary view or grid view with sorting options were also highly valued. Among the representation views that were considered the most intuitive by users were the comparison of the situation of a particular socio-demographic group for several countries and the visualisation of inconsistencies, as they favour decision-making and save users time and effort.

### (Phase II) with non-state actors

**Figure 6:**
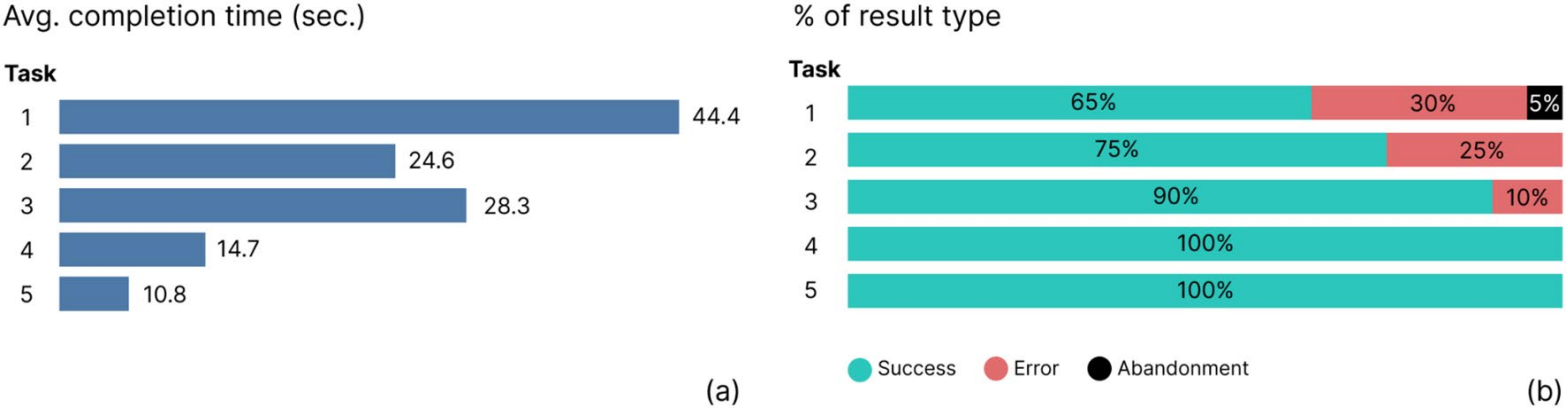
Completion time for the usability test (**a**) and result rates (**b**).

User testing with non-state actors showed the following results for each of the tasks: **Task 1** (*General overview with country information*) was the task with the highest error (30%) and abandonment rates (10%), and the highest completion time (44.4 s). For **Task 2** (*Detailed view by country and socio-demographic group*), we obtained 24.5 s for completion time and 25% of error rate. **Task 3** (*Socio-demographic group comparison view*) presented 28.3 s for completion time and 10% of error rate. **Tasks 4 and 5** (*Analysis of inconsistencies and Summary view*) had very short completion times (14.7 and 10.8 s respectivaley) and 100% success rate (see Fig. 6).

**Figure 7:**
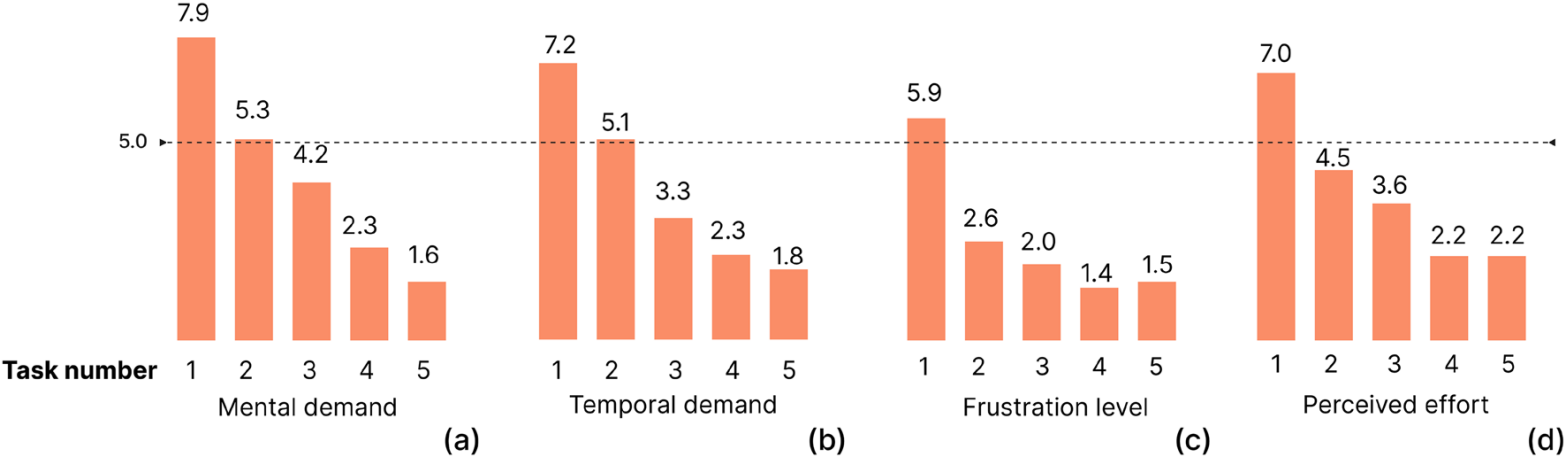
Average values of questionnaire results on perceived difficulty (based on NASA-TLX categories) for the five tasks and for different metrics, from Mental demand (**a**) to Perceived effort (**d**). For each metric, a higher value implies major problems during the resolution of the task.

From the NASA-TLX results, we identified the level of visualisation difficulty linked to each of the tasks, based on the different metrics involved (see Fig. 7). The general overview visualisation (Task 1) required a high level of mental demand, temporal demand, frustration and perceived effort from the participating non-state actors presenting values over five for all metrics. For the task involving a detailed view by country (Task 2), the cognitive load was moderate, and it was low for the last three tasks, since values for all the selected metrics were under 5. We will analyse these results in the Discussion section together with the comments from users and the design decisions adopted to improve these issues (See also Appendix C.1 and C.2 of the Supplemental Material).

The Bipolar laddering identified positive and negative aspects of the tool: Positive aspects amounted to 63, with a mean score of 8.4, and negative aspects 29, with a lower mean score of 4.6 (see more details in Table 1). While for positive aspects the higher values are better, for negative aspects lower values indicate less severity.

Among the negative aspects, two stood out given their frequency (mentioned 7 and 6 times) and their corresponding level of severity (8.2 and 4.4 respectively). Such aspects were the difficulty to interpret the general overview visualisation showing average opinions by country (see Fig. 3a) and the need to improve some aspects of the legends to make them more intuitive and understandable. In the case of positive aspects, the most highly valued were the flexibility and the high level of customisation of the tool to analyse socio-demographic opinion as well as its simplicity in representing inconsistencies (see Table 1).

## Improvements applied based on evaluation results

From the two evaluation phases we implemented improvements to the first version of the prototype (see section “Tool functionalities”). These improvements were based on quantitative and qualitative metrics gathered during the cognitive walkthrough (with potential users and experts) and the User Testing, Bipolar Laddering, as well as the results from the NASA-TLX questionnaires (with non-state actors).

**Figure 8:**
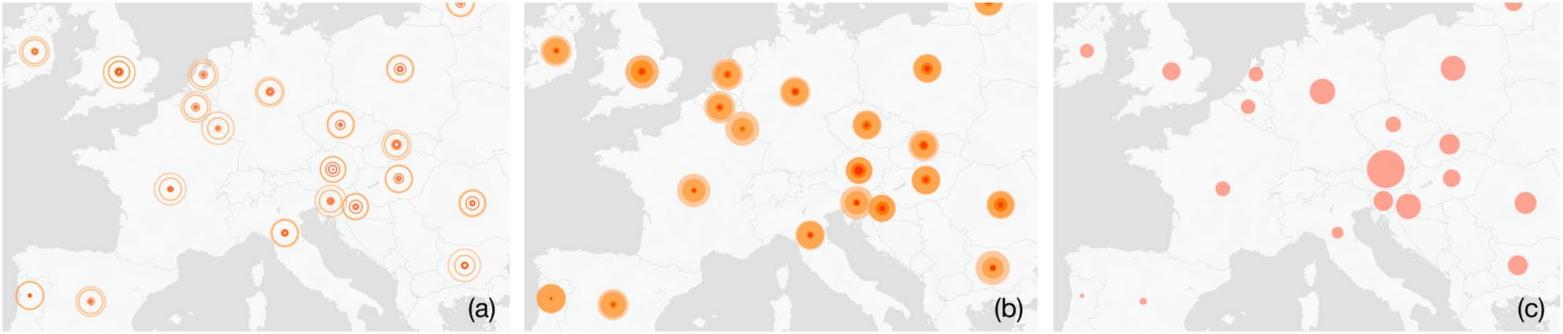
Initial view of the tool through three possible representations for the selected variable: Concentric circles proposed before the user evaluation phase that were found to be difficult to interpret (**a**). Alternative proposal employing concentric areas, after the user test phase (**b**). Options (**a**) and (**b**) present the response percentages for all opinions simultaneously. Bubble map representing just one opinion per country ('Not at all important'), which was also suggested during the evaluation sessions (**c**).

The main improvements carried out were the following:

1. Simplify the general view based on the comments of the user evaluation. (See Fig. 8)
2. Improve the contrast between positive and negative aspects for the detailed glyphs (See Fig. 5) in order to identify the most or least committed socio-demographic groups.
3. Improve and simplify some of the legends for the comparing view.
4. Simplify interaction to navigate from the general view to the comparison view (See Fig. 3).
5. Allow access to the specific metrics for each socio-demographic group, even those not presenting any kind of inconsistencies or anomalies.

The modifications in this version stem from comprehensive user feedback and the outcomes obtained through the evaluation phases (Phase I and II). The revisions were meticulously crafted based on the insights gathered from users' experiences and the detailed assessments conducted across both evaluation phases. These adjustments aim to address the identified concerns and enhance the overall user experience in alignment with the valuable input received from the evaluation processes. These modifications are discussed in more detail in the next section.

## Discussion

Service design allows products and services to be designed for target users and address the needs of final customers. In this work, we design, optimise and align the operations of administrative institutions to better support the needs of citizens, enhancing the cooperation between different administration’s departments and implementing tools offering true value and solutions to enhance the design and definition of better policies. In this section we will discuss the main steps of the methodology and the impacts on the final tool, by simplifying interaction, making access to information more intuitive and favouring comparison and highlighting relevant issues in citizens’ attitudes and habits.

### The importance of evaluating the tool from different perspectives

We used service design to identify potential users' needs and the main questions to be answered with the data available. Through the design process, we also discovered the value of how a high level of customisation in the visualisations can affect cognitive aspects and favour decision-making processes. In this respect, the user interface design can be a strategic step, giving us a “big picture” of the challenge^45,52,67,68^. The service design process also gives methodologies to redefine how problems can be tackled, identifying opportunities and barriers and offering broader solutions, always from a user-centred design approach and also letting the user play an important role in the co-creation.

The final evaluation with non-state actors was meant to verify whether the requirements, functionalities and design choices made sense beyond those made by potential users and experts. Clearly, most of the proposed solutions would meet the needs of potential users, as they themselves participated in the co-creation sessions. Similarly, experts gave their point of view on problems that potential users and non-expert audiences may not have detected previously^57^.

For this reason, the test with the non-state actors ensures that the final design of the tool is suitable for an audience with similar needs but without expert knowledge in data visualisation^69–71^.

### Simplifying interaction, acting as visual information architecture

The global view was the most difficult representation for both expert users and non-state actors alike. Figure 8a,b, show a general view representation proposed by data visualisation experts, which favours comparison against other alternatives and effectively addresses the overlap problem. However, this type of visualisation was not familiar to any of the audiences involved in the study. An alternative solution arose from the validation process, in which instead of visualising all responses at the same time, users could select the category they were interested in (i.e. ‘Very important’, ‘Strongly agree’, etc.). This visualisation allowed us to simplify the view while keeping a coherent transition to a more detailed view by country (see Fig. 8c).

Graphs showing a detailed view by country, although looking complex at first sight (Fig. 5), were flexibly adapted to the changing questions to be answered through the visualisation. Sometimes potential users need to know the least committed groups in order to target them more effectively (See Fig. 5a). Other times they need to identify the most committed groups (See Fig. 5c), or groups in the same category (See Fig. 5b) to study the reasons why policies are working effectively for them. In terms of interaction, the user can choose which countries to view and at what zoom level. This avoids glyph overlap problems on the map and allows users to determine how much information they want to compare at any given time. Thus, we avoid the cognitive overload of our audience while providing them with a greater sense of control.

In terms of usefulness, users highlighted the sheer amount of information presented in the glyphs with social group details (see Figs. 3b,c and 5), but stated that it was presented in a gradual and controlled manner: *“As you explore, you can hide and show the opinion in detail by groups, for the countries of your interest. There is a lot of information, but at all times you can control what you want or do not want to see”.* However, other participants needed a brief time for analysis because they were not familiar with the representation: *“The breakdown by* socio-demographic *group/country is surprising the first time you access the information. There is a lot of information. However, once you understand the distribution of the circular plots, it is very easy to understand. I can not think of a better map representation, as it optimises the space very well through the proposed interaction".* In this sense, the onboarding process is needed and helps to improve the user experience for novice users.

It should be pointed out that data visualisation experts in particular, complained about the use of more intense colours to represent lower values (‘Totally disagree´), given that best practice visualisation rules dictate the opposite^72^. This design decision (which was obvious to potential users as they had established this requirement) as sometimes they need to highlight the least committed groups and countries, as these are the ones to be targeted by environmental awareness policies and campaigns. However, the tool also makes it possible to highlight the most environmentally committed groups (see Fig. 3c). In this case, potential users had stated that this functionality was also necessary to be able to investigate the reasons why certain policies and social criteria are successful for specific groups.

Both experts and non-state actors valued the possibility of comparing the level of commitment of a certain socio-demographic group for different countries (see Fig. 4a). The participants stated that this functionality, as well as the type of representation used, was very useful. Once they had become familiar with it, they found it very clear and intuitive. However, this task (as well as the previous one) required a slight learning process the first time they accessed the visualisation. This option was a result of the need of potential users to compare the environmental response for a given group in different regions (countries in the case of the Eurobarometer). For this task, the adaptation period was limited to the interpretation of the legend. In this case, the users stated that the arrangement of the groups (clockwise direction according to the level of commitment) was clear and intuitive, once they understood how to interpret the graph. One of the users explained: *“You just have to read the legend to interpret the graph. In this case using clockwise movement, it is very intuitive and easy to understand."*

Visualisation of inconsistencies (groups that express great interest in certain environmental problems, but which do not take action to mitigate them) was considered one of the main features of the tool (see Figs. 2.8 and 3c). Non-state actors and experts praised the clear and intuitive way in which the information was presented. One of the participants declared: *“I think it is one of the most powerful aspects. It presents and compares information from several variables and contrasts intention and action. With my current tool, it would take at least an hour to collect the data. Here I have the information in one click”. “An important aspect is that you have access to the most common channels of communication, that is to say, which type of media they consume, newspapers, TV, social networks, so you don’t only know about their main concerns but the channels you need to use to address a specific group”.*

Potential users highlighted the importance of this functionality as it greatly facilitates decision making and visually explains the barriers faced by the least committed groups. A comparative task that would require consulting different variables is obtained automatically with a simple click. In addition, the view of inconsistencies provides information on the media and information channels frequented by a given group. One of the participants stated that *"This will help define specific policies for specific attitudes. Target groups with more optimal advertising campaigns, empathising with their current problems to motivate a change in their attitudes”.*

Furthermore, we detected a possible new functionality which could be included in the configuration area: Establish the thresholds or percentages from which a particular behaviour is to be considered an inconsistency. Currently, interest above 50% and action below 25% are considered to be an inconsistency. An advanced user could, for example, modify these thresholds according to their particular needs at the time. This would have a direct impact on the number of inconsistencies to be displayed.

The summary views were another of the most valued visualisations, in particular by the management profiles, as they allowed for the analysis of the most salient information (See Fig. 4b,c). They are also useful as they can be incorporated into printed reports. One user declared that *“It shows, in a very direct way, the most relevant things to take into account, less committed groups from different countries. It does all the work for you and also allows you to select what you want to highlight”. “This pilot (referring to the tool) is focused on environmental opinion, but it can be used in the same effective way for any other social issue.”*

Although the tool has a basic navigation (main menu and configuration panel), the entire hierarchy of information (global view by country, comparative socio-demographic view, comparative view at country-group level and detailed view of anomalies) is based on the visualisation itself and its associated interaction. We could then speak of the term Graphics architecture or visualisation architecture. In our concrete example a top-down architecture that allows us to navigate through the visualisation from the most general to the most detailed information^73,74^. In most cases, the main navigation (external to the visualisations) allows the users to access each of the sections or views, but in the case of *Op-e-nion*, the external navigation is only acting as an auxiliary element configuring how we show the information rather than what information we want to show.

### The controversy surrounding the use of maps in decision making tools

The visualisation of information on maps facilitates the analysis of complex data in a graphical and easy to understand way, providing a more realistic vision of the conditions that must be evaluated. Therefore, maps are used in various fields such as public administration, and in the administration of transport, infrastructure, health, or social services^75^. From a data visualisation perspective, experts questioned the use of maps, thus favouring the approach used in the summary view. However, potential users preferred maps, especially in a municipal context, since they provide well known geographic metaphors and offer more freedom to explore and compare nearby or adjacent areas. Despite the challenges that maps present at the spatial level for representing multi-categorical data, they are very familiar for the audience and solve the geographic component in a natural way^10^.

### The importance of flexibility and customisation in decision making

In addition to the validations carried out by expert users and non-state actors, tests with potential users showed that the tool’s high level of customisation allowed them to focus on different aspects while prioritising the relevant information at the same time. One of the potential users declared: *“The tool allowed me to highlight what I needed at all times. When I visualise opinions, for example about concern for the environment, I can highlight the most positive or the most negative responses, and the visualisation totally changes its appearance. This personalisation helps me understand what I need at all times".* This requirement was due to the fact that sometimes users also need to highlight the most committed groups as they need to analyse the current conditions to obtain information on the policies that have greater acceptance from citizens in certain areas.

The tool allows for information comprehension through the use of different sorting options based on user needs, highlighting certain values ​​over others, simplifying the way users analyse and compare socio-demographic group inconsistencies and attitudes, and favouring understanding and decision making. Making the necessary information available and in the appropriate format for the development of the task, reduces the sensation of frustration and perceived effort, increasing the level of confidence. Likewise, the flexibility to use sequential or divergent scales was well received, as it helps users analyse differences between extreme positive and negative answers.

### The role of potential users in the prototype definition

The tool presented is a prototype focused on solving the needs of a specific user group. However, scalability aspects were taken into account, considering the possibility of including new variables to expand the information to be displayed and tailor it to the needs of other users. Besides, the data currently displayed in the prototype, gathered from the Eurobarometer, contains total information by socio-demographic group but lacks other levels of aggregation that could also be useful (i.e. we know the percentage of engaged students, but not what percentage of them belong to the rest of the categories).

With the implementation of the final tool, the inclusion of metrics systems to identify the most used views or the time to complete certain tasks is recommended, since it will allow to identify new functionalities or improve the existing ones.

### Limitations

Our research primarily centres on the development of tailored interactive visualisations for survey datasets. While this approach yields valuable insights into data exploration and presentation, it is important to acknowledge certain limitations: The exclusive reliance on visualisation-driven analysis might constrain the depth of insights that can be derived from complex datasets. By focusing primarily on visual exploration, there is a potential limitation in uncovering more intricate patterns or correlations that could be revealed through complementary computational techniques like statistical modelling, data mining, or AI.

Future iterations of this research might consider augmenting the visualisation-centric approach with predictive analytics or machine learning methodologies. This integration would enable a more holistic analysis, potentially revealing deeper insights and patterns within the surveyed data.

With regards to the iterations recommended in Agile methodology, while two iterative prototypes were developed, the acknowledgment of the potential benefits from an additional round of co-creation to further refine the tool and address additional user needs is noted. It is imperative to highlight that these prototypes represent a Minimum Viable Product (MVP) and serve as a foundational step, recognizing the ongoing need for refinement and enhancement to better meet the evolving user requirements.

Please note that due to the confidential nature of our tool and its status as an in-development project, direct access and testing by readers are not available. We appreciate your understanding of these limitations, and we have provided comprehensive details, visualisations, and references to ensure a thorough understanding of the tool's functionalities and design.

## Conclusions and lessons learnt

Public opinion datasets often contain information on different socio-demographic groups and multiple variables. This high-density information requires advanced visualisation tools to understand patterns and trends in data. However, the visualisation tools currently available to organisations and institutions are complex and not necessarily flexible, so it is necessary to resort to tailor made tools.

The results obtained through service design methodology indicate the usefulness of this methodology to co-create visual tools adapted to specific user needs^52^. The validation phase allows for the identification of problems that can be solved in the early stages of the design. Then, the double review process allows for the evaluation of the tool from various points of view: First, from a functional perspective, where potential users and experts identify problems at the visualisation and user experience levels. Second, from an applicability and usability perspective, where common tasks are evaluated by non-state actors to guarantee that the proposed solution would be user-friendly for a broader public.

Tools that allow for the analysis of citizen opinion, not only at a general level but also in relation to the opinions and behaviours of different socio-demographic groups, are key to understanding social attitudes. Therefore, they are a valuable asset for public institutions and administrations to deal with the problems and barriers faced by each socio-demographic group, understand their (in)action and ultimately design more suitable and personalised campaigns to promote action.

The design of visualisations that favour the understanding of complex, multi-categorical data, is a challenge for different fields, especially when the visualisation targets non-scientific audiences without a solid knowledge in data visualisation. In the case of multiple user profile scenarios with very diverse needs, the application of a user-centred design focusing solely on the most common functionalities or requirements would not be sufficient. The customisation of the tool must take on a new dimension and not limit itself to a basic level of analysis, but rather offer high flexibility to meet specific and sometimes evolving needs^39^.

The main lessons learnt during the design process of the tool described in the present study can be applied to any other service aiming to facilitate the analysis and visualisation of public opinion data of any kind to various types of users, independently of their level of expertise in dealing with multi-categorical datasets. These learnings are summarised in the points 1–5 below.

1. Understanding the concerns, actions taken, and information channels used by different socio-demographic groups, can be a first step to delve into the true reasons why citizens do not actively engage in the activities promoted by certain campaigns. Being aware of factors such as precarious economic situations or vested interests, can help public servants understand different behaviours and tackle social barriers from a new perspective.
2. Including a validation with experts and non-state actors as part of the user-centred design is helpful to assess the point of view of potential users, which are often overly focused on their own particular needs and can miss some general usability aspects. This is a valuable new step that has been introduced into the methodology, as the detection of problems when using a tool can lead us to redefine and improve the initial approach and, at the same time, enhance the sustainability of the final product development^76^.
3. Visualising multi-categorical data must be easy and flexible, and adapt to the specific and changing needs of potential users (e.g. administrations and public institutions). In these cases, generalising functionalities that are suitable for the majority of users is not enough, and it is necessary to combine usability with a high level of customisation. In other words, users should be able to see the information in a way that facilitates the task they want to perform at a given time, and according to their personal needs.
4. Different visualisations can target various profiles and objectives within the staff of administration and are closely related to the search patterns of users. Thus the visualisations need to be tailored to support both exploration and analytical tasks (used by analysts, data experts, sociologists, etc.) as well as management tasks aimed at obtaining summaries, rankings and information for reaching conclusions.
5. Providing powerful functionalities can increase the level of demand from users. Indeed, advanced features allow users to make better use of the tool and identify additional needs which, in turn, lead to new features. This is an iterative process which promotes constant improvement.

Facilitating the analysis of public opinion information that supports decision-making will help administrative bodies to develop more efficient and group-centric campaigns. However, administrations and public institutions will not be the only beneficiaries, since this knowledge will be used to support the development of policies truly shaped by the needs of citizens. This will contribute to a paradigm shift: helping to empower citizens and make them more responsible and aware of the need to change habits and, at the same time, urging administrations to implement the necessary measures to deal with the social and environmental challenges that we are now facing.

### Supplementary Information

Supplementary Information.
