# Supplementary material for: Analysing environmental opinion using highly customisable visualisation tools to understand citizens’ attitudes and barriers
Source: Sci Rep. 2024 Feb 16;14:3939. doi: 10.1038/s41598-024-54457-3 (PMC10873361; doi:10.1038/s41598-024-54457-3)
Supplement: Supplementary file 1 — Supplementary Information. [file 41598_2024_54457_MOESM1_ESM.pdf]

Appendixes

Appendix A. Detailed Eurobarometer description

Eurobarometer is a series of open datasets published by the European Union on public opinion on different topics of public interest. Specifically, our dataset referred to the opinions of EU citizens on environmental issues collected in 2017. The data is presented in Excel format (.xls), with one file for each member state. Each file contains several tabs, with information related to a specific question on the environment. There are forty-one survey questions (or tabs) in total, showing the total rates for each country but also broken down into thirty-six different socio-demographic groups (see Section A.3 of the Appendix).
The tools currently available to users of Eurobarometer only allow for the visualisation of the answers by country at a global level, but not for the exploration of the answers at a socio-demographic level (See exploration charts in Fig.1 of this Appendix) .

These socio-demographic groups are based on certain characteristics of the population (gender, age, family situation, employment situation, place of residence, etc.).
We are therefore faced with multivariate/multidimensional data (answers to several questions) which is also multi-categorical. The categorization aspect in this case is double: On the one hand the thirty-six categories themselves, and on the other hand, the five possible answers based on two Likert scale possible answers: ('Very important', 'Fairly important', 'Not too important', 'Not at all important', 'Don't know' or in other cases with 'Strongly agree', 'Quite agree', 'Disagree', 'Totally disagree' and 'Don't know').

The 'Don't know' category (which some users considered the most negative and others considered neutral) retains its negative character, and is located in the centre of the scale, with medium opacity.
Some of the questions posed, referred to the level of importance given to the environment by the respondents, or which aspects were of greatest interest or concern to them ('endangered species and ecosystems', 'floods and droughts', 'pollution' or 'climate change'). In turn, respondents were asked about the actions they carry out to cooperate in conserving the environment and with what frequency, the effects on health, and the level of involvement that private companies, institutions, governments and even the European Union itself should have.

The sampling chosen for the Eurobarometer surveys was based on a random selection of points according to the stratification of the resident national population in terms of metropolitan, urban and rural areas, and proportional to the size of the population (to guarantee total coverage of the country) and its population density [1].

The Eurobarometer survey answers were presented in two languages: French and English.

The variables are represented in table A1. We group them by category according to their area of scope (citizen empowerment, impact on health and environment, responsibility and EU policy obligations). Information about the type of answer is also included (See Table A.1). Some other information, distributed in different tabs referred to the lack of actions by citizens or inconsistencies between concerns and actions: For example, some respondents claimed to be concerned about specific environmental issues (pollution, drinking water, plastic production, climate change, etc.), but, in spite of these claims, did not carry out any corrective actions to improve the situation relative to what they were concerned about. They were classified by potential users in ten categories that related concerns expressed and actions with their impact in each category. This information is included in Table A.1 and A.2.

Table A.1 Variables included

**Empowerment**	Type of answer (Very important - Not at all important)
How important is protecting the environment to you personally?	
**Impact**	Type of answer (Totally agree - Totally disagree)
– As an individual, you can play a role in protecting the environment in your country.<br>– Environmental issues have a direct effect on your daily life and health.<br>– You are worried about the impact of everyday products made of plastic / chemicals on your health.<br>– You are worried about the impact of everyday products made of plastic / chemicals on the environment.	
**Responsibility**	Type of answer (Totally agree - Totally disagree)
– The big polluters should be mainly responsible for making good the environmental damage they cause.<br>– In your opinion, is each of the following currently doing too much, about the right amount, or not enough to protect the environment?<br>a) Big companies and industry<br>b) Citizens themselves<br>c) Your region /area<br>d) Your country / National government<br>e) The EU	
**EU Policy Obligations**	Type of answer (Totally agree - Totally disagree)
– EU environmental legislation is necessary for protecting the environment in your country<br>– The EU should be able to check that EU environmental laws are being applied correctly in your country<br>– The EU should assist non-EU countries to improve their environmental standards	

Table A.2 Information to define inconsistencies

**Concerns & Interests**
(1) Decline or extinction of species and habitats, and of natural ecosystems (forests, fertile soils)<br>(2) Shortage of drinking water<br>(3) Frequent droughts or floods<br>(4) Pollution of rivers, lakes and groundwater – Marine pollution<br>(5) Air pollution<br>(6) Noise pollution<br>(7) Climate change<br>(8) Agricultural pollution (use of pesticides, fertilisers, etc.) and soil degradation<br>(9) Plastic production<br>(10) Use of chemicals
**Actions performed**
– Choose a more environmentally-friendly way of travelling (walking, cycling, public transport, electric car) (Related to 5,6,7,3)<br>– Avoid buying over-packaged products (9,4)<br>– Avoid single-use plastic goods other than plastic bags (e.g. plastic cutlery, cups, plates, etc.) or buy reusable plastic products (9,4,1)<br>– Separate most of your waste for recycling (1,4)<br>– Cut down your water consumption (2)<br>– Cut down your energy consumption (e.g. by turning down air conditioning or heating, not leaving appliances on stand-by, buying energy-efficient appliances) (7,5,3)<br>– Buy products marked with an environmental label (10,8)<br>– Buy local products (Related to 7,1,3)<br>– Use your car less by avoiding unnecessary trips, working from home (teleworking), etc. (7,3)
**Level of activity** (actions)   Many - Some - Few - One - None
**Information channels**
– National newspapers<br>– Regional or local newspapers<br>– Magazines<br>– TV News<br>– Radio<br>– Films and documentaries on television<br>– Family, friends, neighbours or colleagues<br>– Books or scientific publications<br>– Brochures or information materials<br>– Events (conferences, fairs, exhibitions, festivals, etc.)<br>– Museums, national or regional parks<br>– Online social networks

– The Internet (other websites, blogs, forums, etc.)

Table A.3 Social categories with socio-demographic groups selected from the Eurobarometer

**Socio-demographic groups**
**Gender:** Men / Women
**Age:** 15-24 / 25-39 / 40-55 / 55+
**Socio-professional category:** Self-employed / Managers / White collar workers / Manual workers / House persons / Unemployed / Retired / Student
**Marital Status:** Married / Single living with a partner / Single / Divorced / Widowed
**Difficulty paying bills:** Always / Frome time to time / Never
**Considered as belonging to:** Working class / Lower-middle class / Middle class / Upper-middle class / Upper class
**Living in:** Rural village / Small town / Large town
**Politics:** Left / Centre / Right
**Internet use:** Everyday / Often / Never

Appendix B – Test environment and Tasks

Below we describe the statements and conditions of each of the tasks presented in the user test.

Table B.1 User testing tasks (Evaluation Phase II with non-state actors).

**Task 1:** Identify the three countries on the map which are less committed to the environment.
**Task 2:** Which are the two least committed socio-demographic groups for Austria with regards to the environment?
**Task 3:** Imagine that we want to compare the level of commitment for the 'Unemployed' socio-demographic group for the countries displayed/expanded. Which country has the least and the most committed Unemployed group?
**Task 4:** Imagine that we want to analyse the particularities of the socio-demographic groups that present incongruences. That is to say citizens that manifest differences between worries and actions to tackle environmental issues. Can you tell which these socio-demographic groups are and the channels of information that they get information from for France?

**Task 5:** Find the way to access the Summary View? Which are the two least committed socio-demographic groups-countries in Europe?

Table B.2 Task statement, success and error definitions, and time measurement description for Task-based questions (quantitative evaluation).

**Task based questions (Quantitative Evaluation)**			
**Task stat.**	**Success**	**Error/failure**	**Time**
**1**	The user correctly identifies the least committed countries: Hungary, Italy and Austria	The user identifies the wrong countries or they mention just one/two of them.	The time is measured from the time the visualisation is presented, to the time the user starts to talk.
**2**	1.The user selects the correct variable to visualise.<br>2. The user locates the country and clicks on the glyph in the global view for the correct country (Austria).<br>3. The user identifies the least committed groups: Unemployed and Upper class.	The user doesn't select the right variable or doesn't locate the country, or does not click on the glyph.<br>The user fails to identify the two least committed groups.	The time is measured from the time the visualisation is presented, to the time the user starts to talk.
**3**	1.The user clicks on the right category group (after interacting with the correct glyph).<br>2. The user identifies the countries with the least and most committed group: Unemployed: France (the least), Hungary (the most).	The user identifies the wrong category or doesn't understand the legend or identifies the wrong countries.	The time is measured from the time the visualisation is presented, to the time the user starts to talk.
**4**	1.The user identifies the dots (using the legend) as the socio-demographic groups with inconsistencies.<br>2. The user reads out the two categories ('Widows' and 'People between 16-25 years old'). The user identifies the communication channels: TV, Documentaries and Newspapers.	The user wrongly identifies the country, the groups or doesn't click on the dots presenting the inconsistencies, or the user doesn't identify the right communication channels.	The time is measured from the time the visualisation is presented, to the time the user finishes talking.*<br><br>** Being a two-step task, sometimes the user could forget the second part, this is when the moderator asks the user*

			to complete the second step of the task
**5**	1.The user locate the summary view 2. The user identifies the two most committed countries/groups: Austria, Latvia in both cases belonging to the 'Working class' socio-demographic groups-countries.	The user doesn't locate the summary view or doesn't identify the right countries-groups.	The time is measured from the time the visualisation is presented, to the time the user starts to talk.

B.1 Participants of the Cognitive Walkthrough (CW)

The participants were drawn from two different participant profiles: The first group profile consisted of five potential users (three men and two women) between the ages of 42 and 56. The second group profile consisted of five experts in the following fields: Data visualisation, two men aged 36 and 46, User experience (UX) two women aged 45 and 39, and Graphic design, one woman aged 28. Neither group received any remuneration/compensation for their participation.

B.2 Conditions of the CW

Prior to the test, we carried out a short context introduction session [2,3] where the participants filled out a standard bioethics form to confirm and accept their voluntary contribution to the research.
Participants were also informed that their questions, actions and comments would be collected. The study was performed in person using a laptop (MacBook Pro 13-inch, 2017, 3.1 GHz and 16 GB RAM) and connected to a 27'' 4K BenQ display (3840x2160). The users freely interacted with the tool (they were not required to perform any set task) and could explore the different views and visualisations.

B.3 Test participants (Non-state actors)

For this study we used a convenience sample of 20 individuals, comprising 9 men and 11 women between the ages of 25 and 62. All the participants were familiar with environmental policy design, but none of them had deep knowledge of advanced visualisation tools. The participants were non-state actors (NsAs) which comprised members of NGOs, biodiversity firms and epistemic communities. Prior to the test sessions, users were asked not to have consumed stimulant substances that could have enhanced or distorted the test performance as well as the test results and cognitive load perception metrics.
The test sessions were carried out over a three-week period. All participants completed the experiment and no data was deleted. The participants did not receive any remuneration/compensation for their participation.

B.4 Context and pilot test

Prior to the non-state actors test, we carried out a short context introduction session [2,3] where each of the participants filled out a standard bioethics form to confirm and accept their voluntary participation in the research. They were also informed of the purpose of the tool and the main goals of the different views. The participants also completed a short questionnaire with details on their age, level of visualisation expertise, experience, skills and confirmed that none of them suffered from colour blindness.

Moreover, a pilot testing session was also carried out with an individual under the same conditions presented to the rest of the users. This pilot test was conducted in order to detect potential problems related to the design of the test itself [4,5]. The results of this pilot did not form part of the final study results.

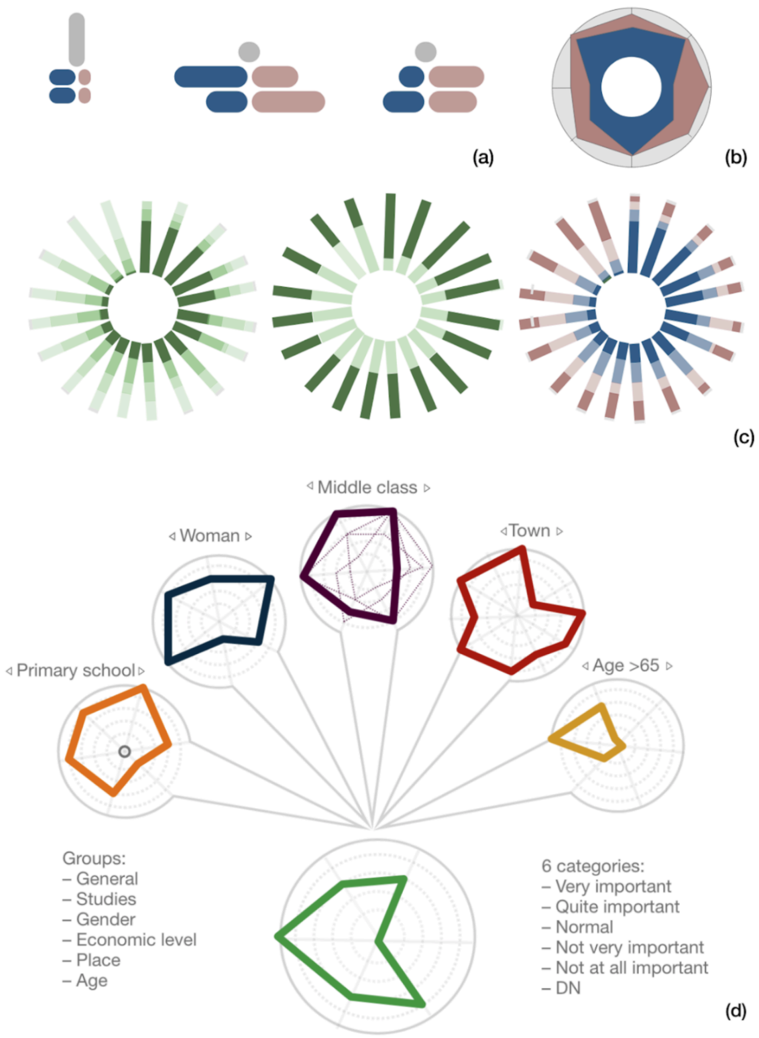

Figure 1: A selection of sample designs considered during the conceptualization phase.

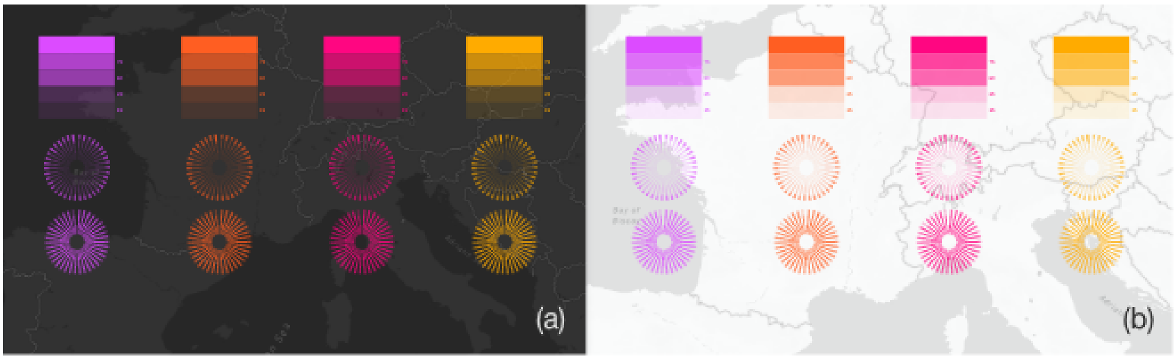

Figure 2: Colours used for the different variables available in the tool: (a) Dark background. (b) Light background. All of them were specifically designed to avoid problems with colour blind users.

B.5 Test environment

We developed a simple online prototype using Axure RP10, to present participants with a very environment to the final functional tool while they performed the test. We presented each of the tasks one-by-one. The test was controlled by a moderator who assisted the participants during the tasks. This assistance consisted of answering any questions related to the task statements but not in helping to solve the tasks themselves or giving any clues on the solutions[6]. The sessions were also recorded in order to be able to review the results in terms of quantitative data (time and success rates) and qualitative data (opinions and valuations).

The test was performed remotely using the ZOOM tool by sharing different links to a functional prototype to access the different tasks.
Participants were informed that times and results, together with their comments, would also be collected. The study was performed using a laptop (MacBook Pro 13-inch, 2017, 3.1 GHz and 16 GB RAM).

After having read the statement, and confirming to the moderator that they were ready, the time to perform the task was measured from the moment the visualisation appeared on the screen and until the participant gave the answer (see Table B.2 of this Appendix).

B.6 Task-based questions (Quantitative evaluation)

During the task-based part of the study, the participants answered five questions about different visualisations available in the tool. More details on the task performance are presented in Table B.1 of this Appendix.

The results of each task (‘success’, ‘error’, and ‘drop off’ or ‘abandonment’ rates) as well as the completion times needed to perform each task were gathered. Drop off metrics measured the number of users who abandoned the task without completing it (in this case due to the difficulty of the task)[7].

B.7 Different views and legends presented during the test.

All the visualisations included in the study were represented under the same conditions:
All the maps/glyphs were presented on light backgrounds. The colours used for the test glyphs (radial stacked bars) were tested presenting the glyphs in orange (see Fig. 2 of this Appendix).

- Task 2 includes a legend with information about how to interpret glyphs with socio-demographic information (see Fig. 2 of the Main Manuscript)
- Task 3 includes a legend about how to interpret group angles as level of commitment (see Fig. 5.a).
- Task 4 includes a legend that explains that dots can be interpreted as anomalies in some of the socio-demographic groups (see Fig. 2 in the Main manuscript)

Five categories were established in relation to the Likert scales and were classified as follows: 'Strongly agree', 'Quite agree', 'Disagree', 'Totally disagree' and 'Don't know'.

Appendix C – NASA-TLX and Bipolar Laddering

Below we describe the questionnaire to measure the cognitive load perceived by users when performing the tasks and the Bipolar Laddering results.

Appendix C.1 – NASA-TLX description

NASA-TLX questionnaire categories[8], includes the following aspects related to perceived difficulty and cognitive resource use:

- Mental Demand or level of concentration required.
- Physical Demand *(not applicable for the type of tasks we are dealing with in this study).*
- Temporal Demand *(NASA-TLX).*
- Perceived Effort *(NASA-TLX) or perceived difficulty while performing the task.*
- Frustration Level *(NASA-TLX).*

Appendix C.2 – NASA-TLX based questionnaire results (Qualitative evaluation)

Below, we present the opinion results for the categories of 'Mental demand', 'Temporal demand', 'Perceived effort' and 'Frustration level'.

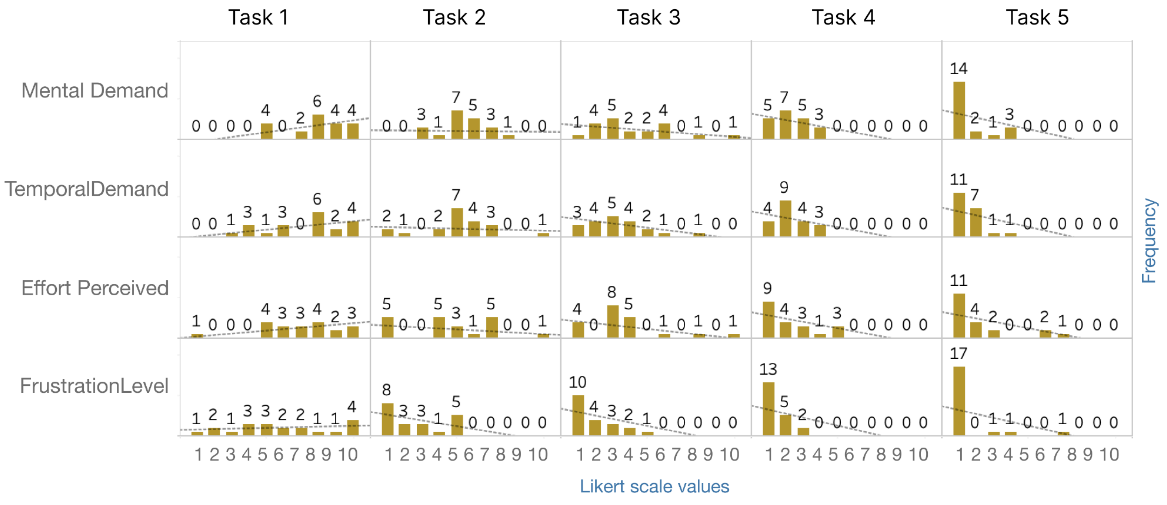

Fig. C.1: Frequency answers to NASA-TLX questionnaire

Using histograms we can analyse the frequencies through which users evaluated the different aspects of each task (see Fig. C.1). We have ignored category 2 (‘Physical demand’), as it was not applicable due to the nature of tasks which did not involve any significant physical effort.

For Task 1, ‘Mental demand’ and ‘Temporal demand’ were the categories with the highest scores with most being valued between 6 and 10. The ‘Perceived effort’ was universally considered to be the most taxing of all the tasks (receiving scores between 5 and 8). For ‘Frustration level’, opinions were spread out over the evaluation spectrum with 10 (very frustrating) being the most voted value with 4 votes.

Task 2 obtained middle-of-the-road values for ‘Mental Demand’ and ‘Temporal Demand’ with 7 votes for level 5 in both categories. However, ‘Perceived effort’ received moderate values, where levels from 1 to 6, were the most voted. Lastly, ‘Frustration level’ was considered low, 1 being the most voted level (with 8 votes) (See Fig. C.1 column 2).

Task 3 presented more defined ‘shapes’ in the distribution, having low-medium values for the categories of 'Mental demand' and ‘Temporal demand', but high values for 'Perceived effort' and 'Frustration Level '(see Fig. C.1 column 3) received low values which indicates that both were considered easy tasks to perform and with a low perceived effort.

Tasks 4 and 5 repeated the pattern of the previous tasks, but with even lower values for the categories of 'Mental demand', 'Time demand', ‘Perceived effort' and 'Frustration level'.
Task 1 obtained high values in ‘Mental Demand’ and ‘Temporal Demand’ and moderate values in ‘Perceived Effort’ and ‘Frustration level’. Tasks 2 and 3 presented moderate values in ‘Mental Effort’, ‘Temporal Demand’ and ‘Perceived effort’. However, the values (level of confidence) were high and ‘Frustration level’ presented low values.

In line with expectations, tasks 4 and 5 presented low values for ‘Mental Demand’, ’Temporal Demand’, ‘Perceived Effort’ and ‘Frustration Level’ (See Fig. C.1).

C.3 Bipolar Laddering and Thinking aloud methodologies (Qualitative evaluation)

After finishing all five tasks, we required the users to review each of them in order to identify the most positive/negative aspects. Once the users had identified these aspects, we asked them to rate each of these aspects from 1 to 10 according to their benefits or severity. The most positive aspects were rated with a 10 (best considered). The most negative aspects were rated as 10 (worst considered or most severe). In this way, Bipolar Laddering allows us to identify the tool’s benefits and barriers, in an effort to obtain the highest values for the positive aspects and the lowest rates for negative answers (as they are considered less important or with a low impact in the users’ perspective[9]. After the Bipolar Laddering technique; users were also encouraged to share their thoughts on the positive or negative aspects of each visualisation, as well as the main difficulties encountered.

Complementing the user testing with other techniques such as Bipolar Laddering, helps to quantify opinions based on their importance. The think aloud technique allows us to understand the reasons behind some user comments [10]. At the same time, these additional techniques have no effect on the quantitative metrics gathered (completion times and success rates) as they are performed after the test.

Appendix D

Table D.1 Visualization Tools based on GIS

Name	Website
QGIS[11]	https://qgis.org/en/site/
GeoPy[12]	https://geopy.readthedocs.io/en/stable/
ArcGIS[13]	https://www.arcgis.com/index.html
MapsBing[14]	https://www.bing.com/maps?cp=41.573334%7E2.110748&lvl=11.0
MapBox[15]	https://www.mapbox.com/
MapTile[6]	https://github.com/michaelstepner/maptile

Bibliography

1. Skaarhoj, K. GESIS - Leibniz Institute for the Social Sciences. https://www.gesis.org/en/eurobarometer-data-service/survey-series/candidate-countries-eb/sampling-fieldwork (1998).
2. Richards, M. Data Visualization and Usability Testing. in (2019).
3. Tomlin, W. C. Putting It All Together: Usability Testing Data Analysis and Recommendations. in *UX Optimization: Combining Behavioral UX and Usability Testing Data to Optimize Websites* (ed. Tomlin, W. C.) 147–175 (Apress, 2018). doi:10.1007/978-1-4842-3867-7_9.
4. Alharbi, A. & Mayhew, P. Users' performance in lab and non-lab enviornments through online usability testing: A case of evaluating the usability of digital academic libraries' websites \textbar IEEE Conference Publication \textbar IEEE Xplore. (2015).
5. Schade, A. Pilot Testing: Getting It Right (Before) the First Time. *Nielsen Norman Group* (2015).
6. Babich, N. Usability Testing: Moderated vs Unmoderated. *Medium* (2020).
7. Finstad, K. The Usability Metric for User Experience. *Interact. Comput.* **22**, 323–327 (2010).
8. Cao, A., Chintamani, K., Pandya, A. & Ellis, R. NASA TLX: Software for assessing subjective mental workload. *Behav. Res. Methods* **41**, 113–7 (2009).
9. Hassenzahl, M. & Sandweg, N. From mental effort to perceived usability: transforming experiences into summary assessments. in *CHI '04 Extended Abstracts on Human Factors in Computing Systems* 1283–1286 (Association for Computing Machinery, 2004). doi:10.1145/985921.986044.
10. Li, Y. & Hollender, N. TASK SEQUENCE EFFECTS IN USABILITY TESTS. 9.
11. Bienvenido al proyecto QGIS! https://qgis.org/es/site/.
12. Welcome to GeoPy's documentation! — GeoPy 2.4.1 documentation. https://geopy.readthedocs.io/en/stable/.
13. Inicio de sesión de cuenta - ArcGIS Online. https://www.arcgis.com/index.html.

14. Mapas de Bing. *Mapas de Bing* https://www.bing.com/maps?cp=41.573334%7E2.110062&lvl=11.0.

15. Mapbox | Maps, Navigation, Search, and Data. https://www.mapbox.com/.

16. Stepner, M. michaelstepner/maptile. (2023).
